# Metatranscriptomics uncovers diet-driven structural, ecological, and functional adaptations in the rumen microbiome linked to feed efficiency

**DOI:** 10.1093/ismeco/ycaf251

**Published:** 2026-01-03

**Authors:** Limei Lin, André L A Neves, Kim H Ominski, Le Luo Guan

**Affiliations:** Faculty of Land and Food Systems, The University of British Columbia, Vancouver, BC, Canada; Department of Veterinary and Animal Sciences, Faculty of Health and Medical Sciences, University of Copenhagen, Frederiksberg, Denmark; Department of Animal Science, National Centre for Livestock and the Environment (NCLE), University of Manitoba, Winnipeg, MB, Canada; Faculty of Land and Food Systems, The University of British Columbia, Vancouver, BC, Canada

**Keywords:** rumen microbiome, microbial ecology, Metatranscriptomics, feed efficiency, dietary adaptation, carbohydrate-binding module, Angus bulls

## Abstract

The rumen microbiome plays a pivotal role in modulating feed efficiency in ruminants, yet the ecological mechanisms mediating the active interactions among microbial adaptations, dietary inputs, and host feed efficiency within the rumen remain poorly understood. To address this gap, we analyzed 120 metatranscriptomic datasets obtained from 30 purebred Angus bulls (each sampled four times) classified as high-feed-efficiency or low-feed-efficiency based on feed conversion ratio, and fed either forage-based (*n* = 15) or grain-based (*n* = 15) diets. We constructed a comprehensive active gene catalog comprising 1 744 067 non-redundant genes and compiled a reference set of 25 115 ruminant microbial genomes. Using integrated Neutral Community Model analysis and carbohydrate-active enzyme profiling, we examined how ecological processes and functional capacities differed across host phenotypes and diets. Neutral Community Model fits revealed that stochastic processes broadly governed rumen microbial community structures (R^2^ = 0.779 for high-feed-efficiency; R^2^ = 0.781 for low-feed-efficiency). Within the predominantly stochastic processes, however, high-feed-efficiency bulls exhibited strong positive selection for diet-responsive microbial lineages: *Fibrobacter* spp. (positively selected species-level genome bins: 61.3%–76.0%; negatively selected: 0%–1.3%), *Butyrivibrio* spp. (positively selected: 13.3%–46.0%; negatively selected: 1.0%–11.2%) under forage feeding, and UBA1067 spp. (positively selected: 33.3%–48.5%; negatively selected: 0%–8.3%) under grain feeding. These lineages encoded catalytic domains appended with carbohydrate-binding modules, such as tandem carbohydrate-binding modules linked to glycoside hydrolases, thereby enhancing substrate adhesion and degradation. In contrast, low-feed-efficiency bulls showed more random community structures and reduced functional specialization. Therefore, these suggest that cattle hosts with higher feed efficiency promote microbial populations functionally aligned with dietary inputs, a process we define as efficient host-mediated microbial amplification. These findings offer new insight into how ecological assembly and functional adaptation of the microbiome contribute to feed efficiency and lay the foundation for microbiome-informed strategies to enhance ruminant production sustainability.

## Introduction

The rumen microbiome constitutes a complex and dynamic ecosystem that mediates the degradation of diverse dietary substrates, fermenting them into volatile fatty acids (VFAs) and other metabolites that serve as the primary energy source for ruminant hosts [1]. This fermentation process underpins host nutrition and has broad implications for livestock productivity and environmental sustainability [2, 3]. Accumulating evidence highlights the profound influence of diet composition, particularly the ratio of forage to concentrate, on shaping the structure and function of rumen microbial communities, ultimately modulating host nutrient acquisition and energy metabolism [4, 5]. Beyond dietary effects, substantial inter-individual variation exists in cattle feed efficiency, whereby high-feed-efficiency (HFE) animals extract more energy from identical diets than low-feed-efficiency (LFE) counterparts, largely due to differences in rumen microbial composition and functional capacity [6, 7]. These findings highlight the need to unravel the ecological mechanisms underlying rumen microbial organization as a crucial step toward improving feed efficiency and reducing methane emissions arising from enteric fermentation [2].

The assembly and maintenance of the rumen microbial community are governed by ecological processes, which in turn influence community structure, metabolic output, and host phenotype [8]. These ecology processes encompass both deterministic and stochastic mechanisms [9]. Deterministic selection involves environmental factors such as diet composition exerting selective pressure on microbial communities and favoring taxa best adapted to specific substrates, while stochastic processes include random events shaping communities independently of the environmental conditions [10, 11]. Therefore, these ecological mechanisms contribute to microbial adaptation: deterministic selection typically enhances specialization and community stability, while stochasticity fosters diversity but may reduce functional efficiency or coordination [12].

Although previous studies have identified specific microbial taxa and metabolic pathways associated with feed efficiency [13], the relative contributions of deterministic versus stochastic processes to rumen microbial community assembly in HFE and LFE cattle remain poorly defined. This gap limits our understanding of whether higher feed efficiency arises from the deterministic enrichment of functionally adaptive, diet-responsive taxa capable of optimized substrate degradation and nutrient assimilation. Addressing this question requires approaches that can link ecological assembly processes to active microbial function.

Most existing investigations have relied on metagenomics, which captures microbial genetic potential but not real transcriptional activity or metabolic output [14]. In contrast, metatranscriptomics enable high-resolution profiling of actively transcribed genes, offering direct insights into microbial function as modulated by dietary and host-specific factors [15]. With the growing availability of tens of thousands of microbial reference genomes, it is now possible to resolve transcriptional activity at the genome level, providing a powerful way to investigate the functional contributions of specific microbial populations. Despite this potential, metatranscriptomics remains underexplored in studies of ecological assembly, particularly in addressing how deterministic and stochastic forces jointly shape microbial function and community structure. As a genome-resolved snapshot of microbial activity, metatranscriptomic data provide a unique opportunity to integrate ecological theory with microbial function, advancing our understanding of the mechanisms linking community assembly, metabolic specialization, and host feed efficiency.

To address these gaps, the present study investigates the structural, ecological, and functional mechanisms underlying diet-driven microbial adaptations in the rumen of HFE and LFE purebred Angus bulls. Although substantial progress has been made in characterizing the rumen microbiota of dairy cows and beef steers [6, 16], little is known about the ecological principles and functional strategies governing microbial adaptation in breeding bulls, a population with disproportionate influence on herd-level genetic performance. To this end, we hypothesized that: (i) microbial communities in HFE bulls are shaped by stronger deterministic selection under both dietary regimes, resulting in the diet-specific enrichment of functionally specialized taxa; and (ii) these taxa possess enhanced substrate-degrading capabilities mediated by enriched repertoires of carbohydrate-active enzymes (CAZymes), whereas LFE bulls exhibit more stochastic assembly and reduced metabolic responsiveness. To test these hypotheses, we constructed a comprehensive catalog of actively transcribed genes comprising 1 744 067 non-redundant sequences derived from 120 metatranscriptomic datasets of rumen fluid collected from Angus bulls fed either forage- or grain-based diets. We further integrated this dataset with a reference genome collection of 25 115 ruminant microbial genomes and applied a combined framework of Neutral Community Model (NCM) analysis and CAZyme profiling. Through this integrative approach, our study provides new insights into the ecological forces and functional mechanisms that govern microbial adaptation to diet and contribute to variation in host feed efficiency.

## Material and methods

### Experimental design and sample collection

All experimental procedures used in this study were approved by the Animal Care Committee at the University of Manitoba under Animal Use Protocol [F11-034/1], in compliance with the Canadian Council on Animal Care guidelines [17]. Thirty purebred Angus bulls (age: 249 ± 22.6 days; body weight: 316.0 ± 31.6 kg) were randomly assigned to two dietary treatment groups (*n* = 15 per group): (i) forage-based (F group) and (ii) grain-based diets (G group) (Supplementary Fig. S1). The detailed chemical composition of the diets was provided in Supplementary Table S1. The bulls were housed in confinement at the Glenlea Research Station, University of Manitoba. The experimental period spanned 180 days, during which all bulls remained in good health. Feed efficiency was evaluated using feed conversion ratio (FCR), calculated as the ratio of dry matter intake to average daily gain. Daily feed intake and weight gain were recorded using the GrowSafe® feeding system (GrowSafe Systems Ltd., Airdrie, Alberta, Canada). To ensure consistency in FCR rankings, data were collected over two periods (Period 1: Days 1–90; Period 2: Days 90–180). Bulls were subsequently classified into two groups based on FCR across both periods: (i) High-FCR (indicative of low feed efficiency, LFE), consisting of individuals that ranked in the top 50% of FCR in both periods; and (ii) Low-FCR (indicative of high feed efficiency, HFE), consisting of individuals that ranked in the bottom 50% of FCR in both periods (Supplementary Fig. S1). To ensure sample reproducibility, rumen fluid samples were collected from each bull at four time points (Days 1, 80, 100, and 180) using a Geishauser oral probe [18]. At each time point, ~250 ml of rumen fluid was collected, immediately snap-frozen in liquid nitrogen to preserve RNA integrity, and subsequently stored at −80°C until RNA extraction and downstream metatranscriptomic analysis. Although the priori power calculation was not conducted, the study design (30 animals, 15 per dietary group, each sampled at four time points) provided balanced replication and statistical robustness at the animal level. The sample size was determined to maximize design-level power for detecting diet- and efficiency-associated effects while accounting for repeated measures across time points.

### RNA extraction and metatranscriptomic sequencing

Total RNA was extracted from 120 rumen fluid samples following established protocols with slight modifications [19]. Approximately 200 mg of rumen sample was combined with 1.5 ml of TRIzol reagent (pH 4.6; Invitrogen, Carlsbad, CA, USA) in bead tubes for RNA isolation. Samples were homogenized and then centrifuged at 12 000 × *g* for 10 min at 4°C to pellet microbial cells. The supernatant was discarded, and total RNA was purified from the pellet using a modified TRIzol-based acid guanidinium-phenol-chloroform extraction method, incorporating 0.4 ml of chloroform (pH 7.0), 0.3 ml of isopropanol (pH 7.0), and 0.3 ml of a high-salt solution (pH 8.0; 1.2 M sodium acetate, 0.8 M NaCl). RNA yield and integrity were assessed using the Qubit 2.0 Fluorimeter (Invitrogen, Carlsbad, CA, USA) and the Agilent 2100 Bioanalyzer (Agilent Technologies, Santa Clara, CA, USA), respectively. Only RNA exhibiting an RNA Integrity Number exceeding 7.0 were selected for RNA-seq library construction. RNA-Seq libraries were constructed using 100 ng of total RNA from each sample with the TruSeq RNA Sample Prep v2 LS Kit (Illumina, San Diego, CA, USA), enabling a comprehensive capture of the microbial transcriptome [20]. Library quality was evaluated through two consecutive measurements on the Qubit 2.0 Fluorimeter (Invitrogen) for quantification, followed by validation using the Agilent 2200 TapeStation (Agilent Technologies) to confirm library integrity. All libraries were then subjected to 2 × 100 bp paired-end sequencing on the Illumina HiSeq 2000 platform at the Génome Québec Innovation Centre (Montréal, QC, Canada), generating an average of ~5.8 Gb (5,778,945,065 bp) of raw sequencing data per sample.

### Construction of an active gene catalog from the rumen of purebred Angus bulls

To construct an active gene catalog from the rumen microbiome of purebred Angus bulls, raw metatranscriptomic sequencing data were processed to remove adapters and trim low-quality reads using Fastp [21] (v.0.20.1) with the parameters “-detect_adapter_for_pe -q 25 -5 -3 -l 50”. Host-derived sequences (*Bos taurus*, GCF_002263795.2) were subsequently removed by mapping reads to the host genome using BWA-MEM [22] (v.0.7.17). Non-mRNA transcripts were filtered out from the remaining high-quality reads using SortMeRNA [23] (v.4.2.0) to isolate microbial mRNA sequences. The resulting microbial mRNA reads were then individually assembled into contigs using metaSPAdes [24] (v.3.15.4) with default parameters. Open reading frames (ORFs) were predicted from the assembled contigs using Prodigal [25] (v.2.6.3) with the metagenomic mode enabled (“-p meta”). Finally, the predicted ORFs were clustered to remove redundancy using CD-HIT [26] (v.4.8.1) with a sequence identity threshold of 95%.

### Taxonomic and functional annotation of the non-redundant active gene catalog

The non-redundant active gene catalog was subjected to taxonomic and functional annotation to characterize the microbial composition and metabolic potential of the rumen microbiome. Taxonomic assignment was performed using DIAMOND [27] (v.2.0.13; *E*-value threshold <1e-5) with a BLASTP search against the NCBI-NR database. To explore carbohydrate metabolism, protein sequences were annotated against the CAZy database [28] using HMMER [29] (v.3.2.1) with a hidden Markov model for each CAZyme family. The mRNA transcripts from each sample were aligned to the gene catalog using BWA-MEM [22] (v.0.7.17) with a minimum alignment length of 50 bp and a sequence identity threshold of 95%. The mRNA abundance was quantified as read counts [30]. The expression of taxa and CAZymes were then calculated by summing the counts of their corresponding annotated genes, providing a comprehensive profile of the rumen microbial community [31].

### Refining rumen prokaryotic genomes and taxonomic and functional annotation

To enable taxonomic and functional analyses at a finer resolution, we collected a total of 25 115 prokaryotic genomes with medium or high quality (≥50% completeness and <10% contamination) by integrating metagenome-assembled genomes of bacteria and archaea from all available rumen studies [1, 5, 31–40], as well as genomes of cultured isolates from NCBI. Genome quality was assessed using CheckM [41] (v.1.2.1) with the lineage_wf workflow. These genomes were subsequently dereplicated at a 95% average nucleotide identity threshold using dRep [42] (v.3.4.5) with the parameters “-pa 0.9 -sa 0.95 -cm larger” to obtain non-redundant Species-level Genome Bins (SGBs). Taxonomic classification of these SGBs was performed by aligning them to the Genome Taxonomy Database (GTDB, v.220) using GTDB-Tk [43] (v.2.3.2). The activity of each SGB was quantified across 120 metatranscriptomic datasets using CoverM [44] (v.0.6.1), with transcript abundance measured as read counts. A maximum-likelihood phylogenomic tree was constructed using PhyloPhlAn [45] (v.3.0.67), and the resulting tree was visualized with iTOL [46] (v.6). For functional annotation, CAZyme profiles were annotated for high-quality SGBs (completeness >80% and contamination <10%) using dbCAN4 [47], enabling detailed insights into carbohydrate metabolism within the rumen microbiome.

### Assessment of stochastic and deterministic processes in microbial communities

To evaluate the relative contributions of stochastic and deterministic processes in shaping the rumen prokaryotic communities of HFE and LFE bulls, we employed the NCM that is an adaptation of Sloan’s neutral theory tailored for microbial populations [48]. The NCM assumes that community assembly is predominantly driven by stochastic processes (ecological drift and immigration), whereas systematic deviations from model predictions reflect deterministic forces such as environmental selection or niche differentiation [49]. NCM analyses were performed on raw read count matrices of SGBs. To ensure statistical independence, models were fitted separately for each sampling time point (Day 1, 80, 100, and 180) within HFE and LFE groups, treating animals as independent units. For each group and time-point combination, the metacommunity relative abundance of ${\mathrm{SGB}}_i$ was defined as ${p}_i=\frac{\sum_j{a}_{ij}}{\sum_{ij}{a}_{ij}}$, where ${a}_{ij}$ denotes the raw count of ${\mathrm{SGB}}_i$ in animal *j*. Occurrence frequency ${F}_i$ was defined as the prevalence across animals at that time point. The expected occurrence frequency follows Sloan’s formulation: ${F}_i=1-\mathrm{BetaCDF}\left(d;\alpha ={Nmp}_i,\beta = Nm\left(1-{p}_i\right)\right)$, with mean community size *N*, immigration probability *m*, and detection limit *d* = 1/*N*. The effective immigration parameter is reported as *Nm*, which reflects the balance between drift and immigration. Parameters were estimated by nonlinear least squares (Levenberg–Marquardt algorithm). Model goodness of fit was quantified by the coefficient of determination: ${R}^2=1-\frac{\sum_i{\left({F}_i-{\hat{F}}_i\right)}^2}{\sum_i{\left({F}_i-{\overline{F}}_i\right)}^2}$, where ${F}_i$ is the observed frequency, ${\hat{F}}_i$ is the model-predicted frequency, and ${\overline{F}}_i$ is the mean observed frequency. The 95% confidence intervals (CIs) for predicted frequencies were calculated using the Wilson method [50]. To estimate uncertainty in *Nm* and R^2^, we performed animal-level bootstrap resampling (1000 replicates per group–timepoint). After obtaining four independent estimates for each group, random-effects meta-analysis (metafor R package [51], v.4.8.0) was applied to pool *Nm* (on the log scale) and R^2^ (linear scale) across time points. SGBs were classified into three partitions relative to the NCM predictions: the “above partition” (${F}_i$ > Upper CI) indicating positive selection, the “below partition” (${F}_i$ < Lower CI) suggesting negative selection, and the “neutral partition” (Lower CI < ${F}_i$ < Upper CI) reflecting stochastic assembly [52]. In this framework, R^2^ reflects the explanatory power of neutral processes. A higher R^2^ suggests that the neutral model provides a better fit, implying a stronger influence of stochastic processes on community assembly. *Nm* is interpreted as an effective immigration parameter under the neutral model. Inference regarding deterministic processes is primarily based on the proportions of above- and below-neutral taxa, rather than *Nm* alone.

### Statistical analysis

Differences in prokaryotic community structure between the two dietary groups (F vs. G) and between HFE and LFE bulls were assessed at the genus level using Principal Coordinates Analysis (PCoA) based on Aitchison distance (Euclidean distance on centered log-ratio (CLR)-transformed data). Group differences were tested with PERMANOVA (adonis2, 9999 permutations), with Animal treated as a blocking factor to reflect the repeated-measures design. The PERMANOVA R^2^ values together with 95% CIs obtained by stratified block bootstrapping were reported. ANOSIM (999 permutations) was additionally performed as a complementary test. To account for potential dispersion effects, homogeneity of within-group dispersion was evaluated using the betadisper function with permutation tests 999 permutations. PERMANOVA, ANOSIM, and betadisper were implemented using vegan [53] (v.2.6.10), and PCoA was performed with ape [54] (v.5.8.1) in R (v.4.3.1). Differential activity across taxonomic levels (phyla, genera, species) and functional categories (CAZymes) was evaluated using MaAsLin2 [55] (v.1.16.0), which fits multivariable linear mixed-effects models to CLR-transformed microbial abundance profiles. For comparisons between dietary groups, models included Diet and Time as fixed effects and Animal as a random effect to account for repeated measures; for comparisons between feed-efficiency groups, models included feed-efficiency and Time as fixed effects with Animal as a random effect. Differential analysis results are presented as Benjamini–Hochberg false discovery rate (FDR)-adjusted *P*-values (*q*), and CLR-based log_2_ fold changes (log_2_FC, defined as the mean difference in CLR values between groups) with 95% CIs estimated by bootstrap resampling.

## Results

### Construction of an active gene catalog for the bull rumen microbiome

To investigate how rumen microbial features are shaped by dietary inputs and feed efficiency, we constructed a comprehensive active gene catalog based on metatranscriptomic profiling of 120 rumen samples collected from purebred Angus bulls. Animals were assigned to HFE or LFE groups based on FCR and were fed either a F or G diet. Metatranscriptomic sequencing yielded 693.5 gigabytes (Gb) of data, with an average of 5.8 Gb per sample and a total of ~6.9 billion sequencing reads (Supplementary Table S2). Following quality control and removal of contaminants and ribosomal RNA sequences, 33.8 Gb of high-quality mRNA data were retained for downstream analysis. De novo assembly generated 3.2 million contigs (Supplementary Table S2). Subsequent ORF prediction and clustering at a 95% nucleotide identity threshold resulted in a non-redundant catalog of 1 744 067 actively expressed microbial genes, forming a high-resolution catalog of the transcriptionally active rumen microbiome. To further resolve the taxonomic origin and functional potential of these transcripts, we aligned the transcripts to a reference collection of 25 115 ruminant microbial genomes, spanning diverse ruminant hosts. Such genome-resolved mapping approach allows direct linkage of expressed transcripts to specific microbial genomes, thereby facilitating strain-level resolution of transcriptional activity.

### Diet-driven differentiation of rumen active microbial communities

**Figure 1:**
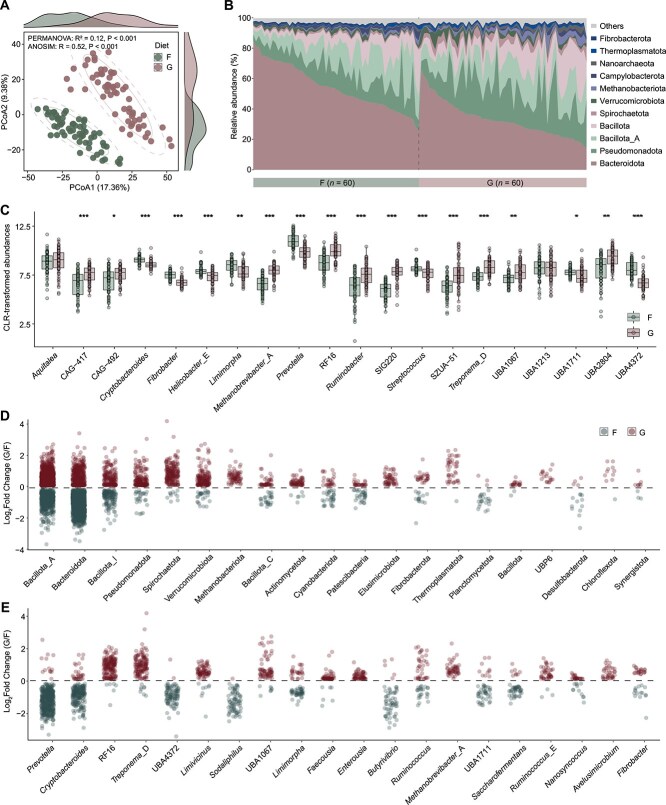
Diet-driven differences in active rumen prokaryotic microbiomes of bulls. (A) PCoA of rumen prokaryotic communities in bulls fed forage-based (F) versus grain-based (G) diets. The solid ellipses represent the 80% CIs, while the dotted ellipses represent the 95% CIs. Group separation was supported by PERMANOVA (R^2^ = 0.124, 95% CI [0.117, 0.144], *P* < .001) and ANOSIM (R = 0.515, *P* < .001). Homogeneity-of-dispersion testing indicated significant differences in within-group dispersion (betadisper, *P* < .001). Sample sizes were F (*n* = 60) and G (*n* = 60), corresponding to 15 bulls × 4 time points in each diet group. (B) Composition of dominant prokaryotic phyla (mean relative abundance >1% in at least one group). (C) Comparison of dominant genera between F and G diets. Values represent CLR-transformed abundances, with boxes showing the interquartile range and center lines the median. (D) Top phyla or (E) genera associated with SGBs exhibiting significant changes in abundance between F and G diets. Significance was assessed by MaAsLin2 (*q < 0.05, **q < 0.01, ***q < 0.001).

To elucidate the impact of diet on rumen microbial community at the transcriptional level, we compared active prokaryotic assemblages between the F and G groups. PCoA based on Aitchison distance (Euclidean distance of CLR-transformed data) revealed clear separation between the two dietary groups, indicating distinct transcriptional profiles of their microbial communities (Fig. 1A). This separation was statistically supported by PERMANOVA (R^2^ = 0.12, 95% CI [0.117, 0.144], *P* < .001) and ANOSIM (R = 0.52, *P* < .001; Fig. 1A), suggesting that diet is a major determinant of microbial activity patterns. Taxonomic profiling at the phylum level showed a community dominated by Bacteroidota (43.9%), Pseudomonadota (16.9%), Bacillota_A (15.1%), Bacillota (10.3%), Spirochaetota (2.6%), Verrucomicrobiota (2.4%), Methanobacteriota (1.6%), and Campylobacterota (1.4%; Fig. 1B). Differential activity analysis revealed significant phylum-level shifts between dietary groups (MaAsLin2, *q* < 0.05; Supplementary Fig. S2). Bacteroidota (log_2_FC = −0.63, 95% CI [−0.84, −0.41], *q* = 0.001), Campylobacterota (log_2_FC = −0.74, 95% CI [−0.95, −0.53], *q* < 0.001), and Fibrobacterota (log_2_FC = −0.62, 95% CI [−0.77, −0.47], *q* < 0.001) showed significantly higher transcriptional activity in the F group. In contrast, Bacillota (log_2_FC = 0.41, 95% CI [0.26, 0.58], *q* = 0.001), Spirochaetota (log_2_FC = 0.94, 95% CI [0.76, 1.14], *q* < 0.001), Methanobacteriota (log_2_FC = 1.26, 95% CI [0.97, 1.55], *q* < 0.001), Nanoarchaeota (log_2_FC = 1.60, 95% CI [1.31, 1.92], *q* < 0.001), and Thermoplasmatota (log_2_FC = 0.87, 95% CI [0.68, 1.04], *q* < 0.001) showed significantly higher transcriptional activity in the G group. At the genus level, significant differences in transcript abundance were also observed (MaAsLin2, *q* < 0.05). Genera such as *Prevotella* (log_2_FC = −1.20, 95% CI [−1.47, −0.92], *q* < 0.001), Cryptobacteroides (log_2_FC = −0.47, 95% CI [−0.60, −0.32], *q* < 0.001), *Limimorpha* (log_2_FC = −0.68, 95% CI [−0.93, −0.43], *q* = 0.006), and *Fibrobacter* (log_2_FC = −0.75, 95% CI [−0.91, −0.60], *q* < 0.001) were significantly more active in the F group, whereas RF16 (log_2_FC = 1.26, 95% CI [0.92, 1.60], *q* < 0.001), UBA2804 (log_2_FC = 1.18, 95% CI [0.78, 1.62], *q* < 0.001), *Treponema*_D (log_2_FC = 1.02, 95% CI [0.78, 1.27], *q* < 0.001), UBA1067 (log_2_FC = 0.72, 95% CI [0.43, 1.03], *q* = 0.003), and *Ruminobacter* (log_2_FC = 1.34, 95% CI [0.88, 1.83], *q* < 0.001) were more active in the G group (Fig. 1C). These taxonomic patterns likely reflect the differing metabolic demands associated with fiber-rich versus starch-rich diets.

To achieve finer taxonomic resolution, we examined non-redundant SGBs, which confirmed and extended the phylum- and genus-level findings. SGBs affiliated with Bacteroidota were more active in the F group, while those from Bacillota_A, Spirochaetota, and Verrucomicrobiota showed higher activity in the G group (MaAsLin2, *q* < 0.05; Fig. 1D). At the genus level, SGBs linked to *Prevotella, Cryptobacteroides*, UBA4372, *Sodaliphilus, Butyrivibrio, Limimorpha*, and *Saccharofermentans* were more active the F group, while SGBs associated with RF16, *Treponema*_D, UBA1067, and *Limivicinus* showed increased activity in the G group (MaAsLin2, *q* < 0.05; Fig. 1E). Together, these results highlight the transcriptional plasticity of the rumen microbiome in response to dietary input. The observed taxonomic shifts likely reflect functional reconfigurations that modulate substrate utilization efficiency and ultimately influence host energy acquisition and feed efficiency.

### Rumen microbial features associated with feed efficiency across diets

**Figure 2:**
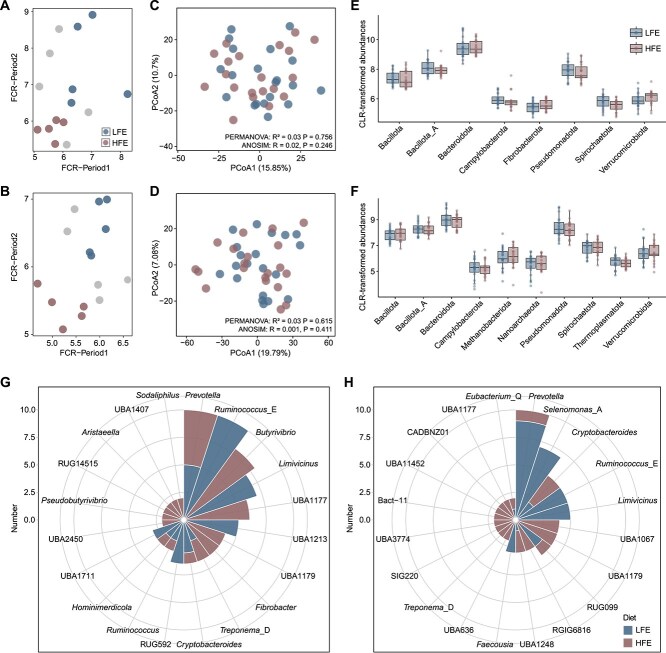
Microbial community shifts associated with feed efficiency across diet types. Rumen microbial composition in bulls with high feed efficiency (HFE, low FCR) versus low feed efficiency (LFE, high FCR) during Period 1 and Period 2 for F (A) and G (B) groups. PCoA based on Aitchison distance (Euclidean distance of CLR-transformed data) of microbial communities in HFE and LFE bulls fed F (C) or G (D) diets. For forage-fed animals, PERMANOVA indicated R^2^ = 0.03 (95% CI [0.027–0.080], *P* = .756) and ANOSIM indicated R = 0.02 (*P* = .246), with homogeneity-of-dispersion testing showing no significant difference (betadisper, *P* = .841). For grain-fed animals, PERMANOVA yielded R^2^ = 0.03 (95% CI [0.028–0.070], *P* = .615) and ANOSIM gave R = 0.001 (*P* = .411), with dispersion testing again nonsignificant (betadisper, *P* = .425). Sample sizes were LFE (*n* = 20) and HFE (*n* = 20) within each diet group, corresponding to 5 bulls × 4 time points in each FE group. Phylum-level compositional differences between HFE and LFE in the F (E) or G (F) groups. Taxonomic distribution of SGBs showing significant differences in activity between HFE and LFE in the F (G) or G (H) groups. Bars indicate the number of differentially active SGBs assigned to each genus. Significance was assessed by MaAsLin2 (*q < 0.05, **q < 0.01, ***q < 0.001).

To elucidate microbial features linked to feed efficiency, purebred Angus bulls receiving either forage- or grain-based diet were stratified into two groups based on FCR across both feeding periods (Days 1–90 and 90–180): HFE (low FCR, bottom 50%) and LFE (high FCR, top 50%) (Fig. 2A and B). Within each diet, five bulls were assigned to each feed-efficiency group and sampled longitudinally at four time points. PCoA, PERMANOVA, and ANOSIM revealed no significant differences in overall rumen microbial community structure between HFE and LFE bulls under either dietary regime (F group: PERMANOVA R^2^ = 0.03, 95% CI [0.027, 0.080], *P* = .756; ANOSIM R = 0.02, *P* = .246; G group: PERMANOVA R^2^ = 0.03, 95% CI [0.028, 0.070], *P* = .615; ANOSIM R = 0.001, *P* = .411; Fig. 2C and D). Consistently, taxonomic profiling showed no significant phylum- or genus-level differences in transcriptional activity between HFE and LFE bulls under either forage- or grain-based diet (MaAsLin2, *q* > 0.05; Fig. 2E and F; Supplementary Fig. S3). These results suggest that feed efficiency in bulls is not primarily driven by large-scale compositional changes in the rumen microbiota, but instead may be shaped by subtle differences in the transcriptional activity of specific microbial taxa.

To resolve microbial activity at finer species-level taxonomic resolution, we mapped transcripts to non-redundant SGBs. In forage-fed HFE bulls, SGBs with elevated transcriptional activity included *Butyrivibrio*, and *Fibrobacter*, whereas those reduced in activity were associated with *Ruminococcus*_E (MaAsLin2, *q* < 0.05; Fig. 2G). In grain-fed HFE bulls, increased activity was observed in SGBs most assigned to UBA1067 and UBA1179, while *Ruminococcus*_E was again more active in LFE animals (MaAsLin2, *q* < 0.05; Fig. 2H). Together, these results suggest that host feed efficiency in beef bulls is governed not by broad restructuring of the rumen microbiota, but by diet-dependent transcriptional modulation of key fibrolytic and fermentative taxa.

### Diet-specific microbial specialization in feed-efficient bulls

**Figure 3:**
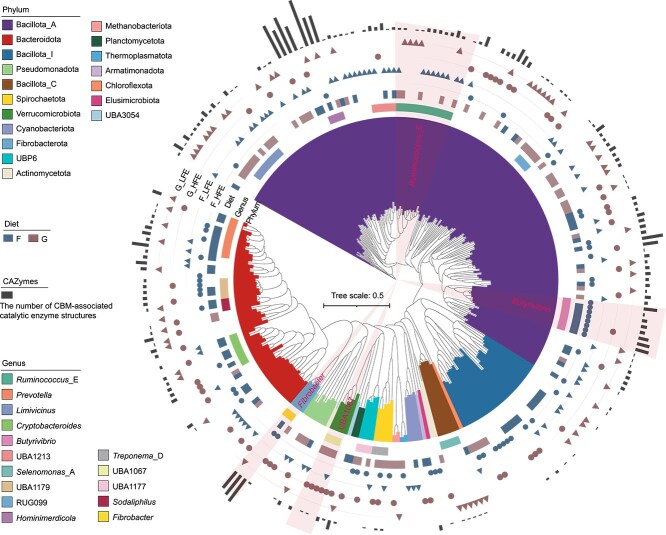
Phylogenetic and functional profiling of rumen SGBs associated with feed efficiency. Phylogenetic tree of 260 high-quality SGBs showing significant relative abundance differences between HFE and LFE bulls. Clades are colored by phylum, with strip charts indicating genus-level affiliations. The heatmap illustrates SGB abundance differences between the F and G groups (blue: higher activity in F; red: higher activity in G). Outer annotations: inner circles for SGBs with higher activity in F_HFE, inner triangles for SGBs with higher activity in F_LFE, outer circles for SGBs with higher activity in G_HFE, outer triangles for SGBs with higher activity in G_LFE. The outermost bar graph quantifies the number of CBM-associated catalytic enzyme structures. Selected representative genera (e.g. *Ruminococcus*_E, *Butyrivibrio, Fibrobacter*, and UBA1067) are highlighted in the tree for clarity.

To further elucidate the interplay between diet and feed efficiency in shaping rumen microbial dynamics, we assessed taxonomic shifts in HFE and LFE bulls within both F and G diet groups. Taxa with increased activity in the F group, such as *Butyrivibrio* spp. and *Fibrobacter* spp., exhibited further higher transcriptional activity in forage-fed HFE bulls (Fig. 3), suggesting a host-driven amplification of fiber-degrading capacity that may enhance energy harvest from cellulose-rich substrates. Likewise, UBA1067 spp., which showed elevated activity in the G group, demonstrated greater activity in grain-fed HFE bulls (Fig. 3), implying enhanced adaptation to starch-rich substrates. These results suggest that bulls with high feed efficiency do not merely respond to diet-driven microbial shifts, but actively promote the enrichment of diet-responsive taxa, potentially amplifying the microbial ability to extract nutrients from specific substrates. However, LFE bulls exhibited limited differentiation in microbial transcriptional profiles across diets. For example, *Ruminococcus*_E spp. remained consistently higher activity in LFE bulls under both forage and grain feeding (Fig. 3), suggesting a relatively fixed microbial configuration with limited ability to adjust to changing substrates. This reduced ecological flexibility may restrict the capacity of LFE microbiomes to optimize fermentation efficiency in the rumen, thereby contributing to poorer feed conversion. Together, these findings support an ecological strategy underpinning feed efficiency in bulls, whereby HFE animals exhibit diet-specific enrichment of specialized taxa such as *Butyrivibrio* spp., *Fibrobacter* spp., and UBA1067 spp., in contrast to LFE bulls whose limited compositional shifts across diets suggest a less flexible microbial community.

### NCM analysis reveals diet-driven selection in high feed efficiency bulls

**Figure 4:**
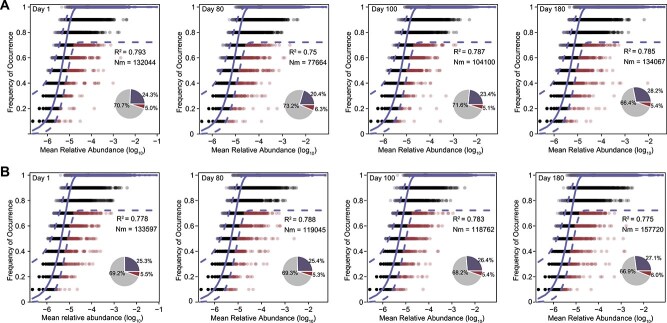
Fit of the neutral community model (NCM) to microbial community assembly in the rumen of HFE and LFE bulls. (A) HFE bulls at four sampling times (Day 1, Day 80, Day 100, and Day 180; *n* = 10 samples per time point). (B) LFE bulls at four sampling times. Solid lines represent the best fit to the NCM, with dashed blue lines indicating 95% CIs around the model predictions. Each point represents an SGB, color-coded by its occurrence frequency relative to NCM predictions: black, neutral partition (within 95% CI), red, below partition (lower than predicted, indicative of negative selection); blue, above partition (higher than predicted, indicative of positive selection). Pie charts show the proportion of SGBs in each partition. The coefficient of determination (R^2^) reflects the variance explained by the model. The effective migration rate (*Nm*) represents the neutral immigration rate.

Given the pronounced enrichment of diet-specific taxa in HFE bulls, this diet-specific enrichment pattern was not observed in LFE counterparts. We hypothesized that the rumen microbiota of HFE individuals may be subject to stronger deterministic selection pressures shaped by diet. To test this, we applied the NCM to transcriptional profiles of SGBs at each sampling time point, pooling estimates across time via random-effects meta-analysis to quantify the balance of stochastic (neutral) and deterministic (selective) processes in microbial community assembly [56]. By assessing deviations from neutral expectations, we evaluated the relative contributions of these processes in shaping the rumen microbiota of HFE and LFE bulls at each sampling time point, with 10 samples per feed-efficiency group. The NCM yielded high coefficients of determination in both groups (R^2^ = 0.779 [95% CI: 0.761–0.797] for HFE; R^2^ = 0.781 [95% CI: 0.766–0.796] for LFE), indicating that neutral processes accounts for the majority of variation in SGB occurrence frequencies in the rumen (Fig. 4A and B) [48, 57, 58]. The estimated migration rate (*Nm*), a proxy for neutral immigration within the NCM framework, was lower in HFE bulls (*Nm* = 108 738 [95% CI 80 745–146 435]; Fig. 4A) than in LFE bulls (*Nm* = 134 435 [95% CI 105 386–171 490]; Fig. 4B). This difference suggests a reduced influence of neutral immigration in HFE bulls, potentially reflecting stronger deterministic filtering. To robustly infer selection, we focused on the proportions of SGBs deviating from neutral expectations (Fig. 4). Within the NCM framework, individual SGBs were classified as neutral, positively selected (above model predictions), or negatively selected (below model predictions). In HFE bulls, 20.4%–28.2% of SGBs were classified as above-neutral (positively selected), 5.0%–6.3% as below-neutral (negatively selected), and 66.4%–73.2% as neutral. In LFE bulls, comparable fractions were observed (above-neutral: 25.3%–27.1%; below-neutral: 5.4%–6.0%; neutral: 66.9%–69.3%). These proportions indicate that while neutral processes dominate rumen community assembly, a consistent subset of SGBs undergoes deterministic selection in both groups (Fig. 4).

We further assessed the selection status of specific taxa enriched in HFE bulls, including *Butyrivibrio* spp., *Fibrobacter* spp., and UBA1067 spp. (Supplementary Fig. S4). For comparison, we also analyzed *Ruminococcus*_E spp., which were more abundant in LFE bulls (Supplementary Fig. S4). *Fibrobacter* exhibited a predominantly positive selection pattern, with 61.3%–76.0% of SGBs classified as positively selected, 24.0%–38.7% as neutral, and only 0%–1.3% as negatively selected. *Butyrivibrio* showed moderate positive selection (13.3%–46.0%), with most SGBs classified as neutral (53.0%–75.5%) and a small fraction under negative selection (1.0%–11.2%). UBA1067 displayed pronounced positive selection, encompassing 33.3%–48.5% of SGBs, alongside comparable proportions of neutral (48.5%–58.3%) and few negatively selected SGBs (0%–8.3%). In contrast, *Ruminococcus*_E in HFE bulls exhibited a largely neutral selection profile, with 70.4%–78.6% of SGBs classified as neutral, 12.6%–23.6% as positively selected, and 4.5%–14.8% as negatively selected, suggesting weaker selective pressure and limited adaptation to efficient rumen environment. These taxon-specific patterns highlight stronger deterministic selection in HFE-enriched bacterial taxa.

### Specific enzymatic mechanisms of diet-dependent taxa with higher activity in HFE bulls

**Figure 5:**
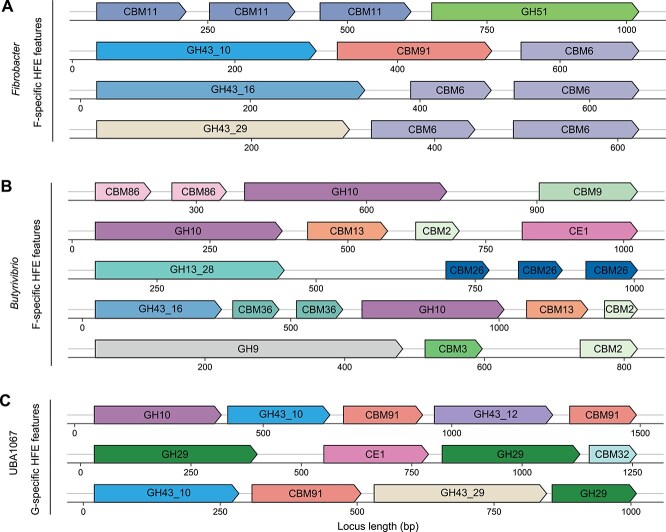
Structural diversity of CBM-binding catalytic domains in rumen microbes of HFE bulls. Schematic representation of tandem CBM structures within catalytic domains of carbohydrate-active enzymes from rumen microbial SGBs in HFE bulls. (A) *Fibrobacter* SGBs with the higher activity in forage-fed HFE bulls. (B) *Butyrivibrio* SGBs with the higher activity in in forage-fed HFE bulls. (C) UBA1067 SGBs with the higher activity in grain-fed HFE bulls.

To elucidate the functional basis for the preferential selection of diet-responsive microbial taxa in HFE bulls, we examined their CAZyme profiles with a focus on CBMs. CBMs are frequently appended to catalytic domains and play a crucial role in enhancing enzyme-substrate interactions, thereby improving catalytic efficiency in the rumen [59–61]. Our analysis revealed that microbial taxa exhibiting elevated transcriptional activity in HFE bulls encoded higher numbers of CBM-associated catalytic domain structures than those enriched in LFE bulls (Fig. 3). Notably, these taxa often encoded multi-catalytic enzymes with tandemly arranged CBMs (Fig. 5), a configuration that markedly enhances substrate binding and degradation capacity [62].

In forage-fed HFE bulls, *Fibrobacter* spp. encoded about 30 CBM-associated catalytic enzyme structures, comprising three tandemly arranged CBMs (e.g. CBM11-CBM11-CBM11) linked to a single glycoside hydrolase domain (GH51) (Fig. 5A). Additionally, *Fibrobacter* spp. harbored multiple CBM6 modules paired with various GH43 subclasses (e.g. GH43_16, GH43_29, GH43_10) and CBM91, facilitating the adhesion and degradation of hemicellulose (Fig. 5A). *Butyrivibrio* spp. encoded a broad spectrum of CBMs targeting cellulose (CBM2, CBM3, CBM9, CBM13), hemicellulose (CBM86), and starch (CBM26), highlighting its metabolic flexibility and its role as a stable contributor to feed efficiency (Fig. 5C). In grain-fed HFE bulls, UBA1067 spp. displayed a rich repertoire of CBMs paired with catalytic domains optimized for diverse carbohydrate substrates (Fig. 5B). These included two CBM91 modules associated with GH43 and GH10 for cellulose and hemicellulose degradation, a CBM32 domain linked with CE1 and GH29 for complex hemicellulose breakdown, and additional CBM91 modules co-occurring with GH43 and GH29, collectively enhancing the degradation of grain-associated polysaccharides. On the other hand, *Ruminococcus*_E spp., which showed consistently lower activity in HFE bulls, encoded significantly fewer CBMs, ~10-fold fewer than its close relative *Ruminococcus* spp. (Fig. 3) [63]. This limited CBM repertoire restricts its ability to adhere to and degrade complex plant substrates, thereby compromising its ecological fitness under the selective pressures of high-efficiency rumen environments. Collectively, these findings underscore the functional convergence of microbial communities in HFE bulls towards enhanced substrate degradation. The enriched CBM-catalytic architectures of key taxa facilitate more efficient utilization of diet-derived polysaccharides, reinforcing their ecological and functional dominance in the rumen.

## Discussion

Our study provides a comprehensive genome-resolved metatranscriptomic analysis of the rumen microbiome in purebred Angus bulls, offering mechanistic insights into how diet and feed efficiency shape microbial ecology and function. By resolving transcriptional activity at the finer level, we constructed a non-redundant active gene catalog comprising 1 744 067 genes, which were mapped to a reference set of 25 115 ruminant microbial genomes. This genome-resolved metatranscriptomic framework goes beyond conventional metagenomic approaches that capture both viable and dormant microorganisms, by specifically profiling actively transcribed genes to reveal the functional potential of metabolically active lineages [15]. Through this approach, we identified distinct genomic and transcriptional signatures associated with diet- and efficiency-dependent adaptations, highlighting how deterministic ecological processes and functional specialization contribute to variation in feed efficiency. Collectively, this active gene catalog establishes a robust foundation for linking microbial gene expression to ecological outcomes, providing a valuable resource for future efforts to decipher how rumen microorganisms respond and adapt to dietary selection pressures.

The transcriptional landscape revealed pronounced plasticity of the rumen microbiome in response to dietary inputs [3, 5]. Taxonomic shifts indicate that the rumen community reorganizes its functional activity to accommodate varying substrate availabilities. Through genome-resolved metatranscriptomics, we also detected transcriptional changes in previously underappreciated, low-abundance lineages such as *Sodaliphilus* spp. and *Limivicinus* spp., underscoring their potential as biomarkers for monitoring microbial responses in precision feeding systems. Notably, these changes were not accompanied by large-scale structural differences between HFE and LFE animals. This suggests that feed efficiency in bulls is governed not by broad compositional restructuring, but rather by fine-tuned transcriptional adjustments within specific lineages. Such patterns align with findings in beef steers, where overall community structure remains largely stable across feed efficiency phenotypes [64, 65], yet diverge from observations in dairy cattle, where pronounced taxonomic shifts have been reported [16]. These contrasts likely reflect production contexts: beef bulls and steers, maintained under relatively stable regimens emphasizing growth and reproduction, promote functional specialization within existing taxa, whereas lactating dairy cows experience frequent dietary transitions and physiological fluctuations associated with lactation-driven microbial instability that drive broader structural reorganization of the rumen microbiome [66].

Within this framework, feed efficiency appears to depend on diet-specific activation of functional groups rather than wholesale changes in microbial diversity [67]. In forage-fed HFE bulls, SGBs affiliated with *Fibrobacter* and *Butyrivibrio* displayed enhanced transcriptional activity, consistent with strong cellulolytic capacity [68]. *Butyrivibrio* spp. are known for its broad carbohydrate-degrading ability and its role in producing butyrate, a key VFA that supports rumen health and host energy supply [40, 69]. In grain-fed HFE bulls, UBA1067 spp. were preferentially activated, reflecting adaptation to starch-rich substrates. Notably, while HFE steers in earlier studies were enriched in *Blautia*, associated with rapid fermentation of simple polysaccharides and VFA production [6], HFE bulls in our study showed enrichment in fibrolytic and butyrate-producing taxa such as *Fibrobacter, Butyrivibrio*, and UBA1067. This divergence underscores sex-specific microbial adaptation: steers may rely more on rapid fermentation pathways for fattening, whereas bulls may favor more efficient degradation of complex substrates to sustain long-term growth and reproduction.

**Figure 6:**
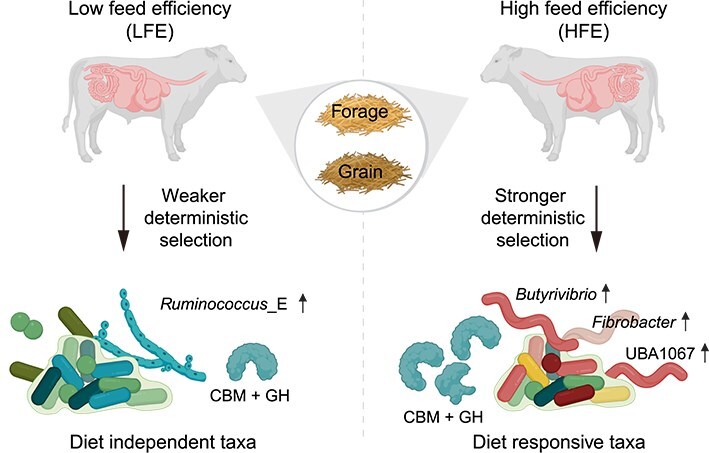
Conceptual model of efficient host-mediated microbial amplification in the rumen. Schematic illustration showing how host feed efficiency shapes microbial community assembly and functional specialization under forage- and grain-based diets. HFE bulls exhibit stronger deterministic selection that amplifies diet-responsive taxa such as *Butyrivibrio* spp., *Fibrobacter* spp., and UBA1067 spp., which encode enriched CBM-associated catalytic architectures that enhance substrate binding and degradation. In contrast, LFE bulls exhibit weaker deterministic selection, resulting in diet-independent microbial configurations dominated by *Ruminococcus*_E, which is characterized by limited CBM-associated catalytic architectures. This framework highlights how efficient hosts reinforce the activity of functionally specialized microbes aligned with dietary substrates. Created in BioRender.

By integrating dietary treatments with feed efficiency status, we observed a striking pattern: taxa that were diet-responsive at the population level exhibited even stronger transcriptional activation in HFE animals. For example, *Butyrivibrio* spp. and *Fibrobacter* spp. were not only enriched in forage-fed bulls but displayed further elevated activity in forage-fed HFE animals, suggesting a host-driven reinforcement of cellulose utilization. Likewise, UBA1067, a lineage responsive to grain-based diets, displayed enhanced transcriptional activity in grain-fed HFE bulls, reflecting improved adaptation to starch-rich substrates. These patterns indicate that efficient hosts do not merely reflect diet-induced microbial shifts but amplify the activation of functionally relevant taxa. In contrast, LFE bulls exhibited more constrained microbial responses, with taxa such as *Ruminococcus*_E maintaining consistently high activity under both diets, suggesting a rigid microbial configuration with limited adaptive flexibility. This reduced ecological plasticity may contribute to the lower nutrient conversion efficiency observed in LFE animals. Building on prior findings that feed efficiency is influenced by diet [65], our findings extend this framework by revealing that host-mediated selection intensifies the activity of diet-responsive microbes, thereby enhancing nutrient extraction in efficient animals. Rather than uniform community-wide shifts, HFE bulls exhibit a targeted enrichment strategy jointly shaped by host and dietary filtering. We therefore propose the concept of efficient host-mediated microbial amplification (Fig. 6), wherein efficient hosts actively reinforce the enrichment of diet-responsive lineages to optimize nutrient release and conversion. This process may be reinforced by host genetic filtering mechanisms, as host genotypes have been shown to influence rumen microbial features associated with feed efficiency [70]. This framework advances beyond compositional descriptions, linking host feed efficiency to selective microbial activation and providing new ecological insights into the mechanisms underpinning ruminant productivity.

NCM analysis further supports the framework of efficient host-mediated microbial amplification by showing that deterministic selection was pronounced in HFE bulls. Although neutral processes broadly govern rumen microbial community assembly [48, 57, 58], HFE animals exhibited a consistently higher proportion of positively selected taxa, particularly *Fibrobacter* spp., *Butyrivibrio* spp., and UBA1067 spp., underscoring stronger diet-driven filtering toward functionally aligned microbes. In contrast, taxa enriched in LFE bulls, such as *Ruminococcus*_E spp., remained largely neutral with limited positive selection, reflecting a less flexible and more neutrally assembled community. More broadly, the balance between stochastic and deterministic processes is a central theme in microbial ecology [71, 72]. In the rumen, both diet and host development have been shown to contribute to deterministic filtering as microbial communities mature [57]. Our results extend this framework by demonstrating that, in mature breeding Angus bulls, deterministic selection exerts a stronger influence in HFE individuals, directing microbial activity toward enhanced substrate-degrading capabilities. This elevated positive selection highlights diet as a major selective force, shaping microbial activity to support efficient energy harvest, while LFE communities remain more neutrally structured and less responsive to dietary variation [13]. Collectively, these findings provide an ecological model in which efficient hosts amplify the activity of diet-responsive taxa, thereby maximizing nutrient conversion efficiency. The observed interplay between stochastic and deterministic processes provides new ecological insights into how diet-microbe-host interactions govern rumen functionality and underpin differences in feed efficiency among cattle [57].

Functional profiling of the CAZyme transcripts further elucidates the mechanistic basis for the diet-driven selection observed in the NCM analysis, revealing how substrate-specific adaptations in HFE bulls enhance energy acquisition. Taxa enriched in HFE animals encoded a greater number of CBM-associated catalytic enzyme structures, frequently featuring tandemly arranged CBMs linked to catalytic domains, which improve substrate adhesion and degradation efficiency [60, 61]. For instance, *Fibrobacter* spp. in forage-fed HFE bulls encoded ~30 CBM-associated catalytic architectures, such as CBM11-CBM11-CBM11-GH51 arrangements, enabling efficient degradation of cellulosic biomass [68, 73]. *Butyrivibrio* spp. in forage-fed HFE bulls encoded CBMs targeting cellulose, hemicellulose, and starch, underscoring its metabolic versatility and role in enhancing feed efficiency [74]. Likewise, UBA1067 spp. in grain-fed HFE bulls encoded diverse CBM-GH combinations capable of degrading cellulose and hemicellulose. These functional adaptations align with the patterns observed in the NCM analysis, wherein taxa possessing enhanced substrate-binding and degradation capacities were under stronger positive selection in HFE animals. Such enzymatic specializations confer a competitive advantage in nutrient acquisition, optimizing microbial metabolism to support host efficiency [7]. In contrast, *Ruminococcus*_E spp. in LFE bulls contained fewer CBMs (~10-fold fewer than a known cellulosome-encoding *Ruminococcus* [63]), limiting its ability to effectively bind and degrade substrates. This functional constraint likely underlies its predominantly neutral assembly pattern, reduced activity across diets, and association with lower feed efficiency. Collectively, these results highlight a functional convergence of microbial communities in HFE bulls toward enhanced carbohydrate degradation. The enrichment of CBM–catalytic architectures among key taxa strengthens their ecological dominance and underscores the molecular underpinnings of efficient substrate utilization in the rumen. This enzymatic specialization provides a mechanistic explanation for the elevated feed conversion efficiency observed in HFE animals and identifies promising candidate taxa and gene families for targeted manipulation in precision livestock nutrition.

In summary, our findings reveal an ecological strategy in which host physiology and diet jointly shape microbial activity. By introducing the concept of efficient host-mediated microbial amplification, we propose a new framework linking host traits to microbial selection, wherein efficient animals preferentially enrich and activate diet-responsive taxa. From an applied perspective, taxa such as *Fibrobacter* spp., *Butyrivibrio* spp., and UBA1067 spp., along with emerging indicators like *Sodaliphilus* spp. and *Limivicinus* spp., represent valuable biomarkers and potential intervention targets for precision feeding and selective breeding. This ecological model advances current understanding by shifting the focus from community composition to functional amplification, offering a foundation for microbiome-informed strategies to improve ruminant productivity and sustainability. Nevertheless, the limited sample size constrains the generalizability of these findings. Future studies incorporating larger animal cohorts, deeper sequencing, and integrative multi-omics approaches will be essential to further validate and refine the proposed framework of efficient host-mediated microbial amplification. Experimental validation through cross-inoculation, in which rumen microbiota from HFE and LFE animals are reciprocally transferred, could directly test host-driven microbial selection and its persistence over time. Longitudinal tracking of these transplanted communities would further elucidate the stability of host–microbiome associations.

## Conclusions

Our genome-resolved metatranscriptome revealed how diet and host feed efficiency jointly shaped the taxonomic and functional landscape of the rumen microbiome in purebred Angus bulls. Feed efficiency was not driven by large-scale shifts in microbial composition, but rather by the transcriptional activation of diet-responsive taxa with specialized metabolic roles. Key lineages such as *Butyrivibrio* spp., *Fibrobacter* spp., and UBA1067 spp. were differentially enriched in HFE animals, highlighting potential microbial targets for precision nutrition and selective breeding. NCM analysis further suggested that efficient hosts exerted strong deterministic pressures to enrich microbial populations functionally aligned with dietary substrates, a process we termed efficient host-mediated microbial amplification. This concept provided a new ecological framework linking host physiology, diet, and microbial function, providing a foundation for microbiome-informed strategies to improve ruminant productivity and sustainability.

## Supplementary Material

ycaf251_Supplemental_Files

## Data Availability

All raw sequencing reads of metatranscriptome have been submitted to the National Center for Biotechnology Information (NCBI) under BioProject accession No. PRJNA1259955. R scripts are available at GitHub: https://github.com/LLMmay/R_Diet_FE.
