# Supplementary material for: Metatranscriptomics uncovers diet-driven structural, ecological, and functional adaptations in the rumen microbiome linked to feed efficiency
Source: ISME Commun. 2026 Jan 3;6(1):ycaf251. doi: 10.1093/ismeco/ycaf251 (PMC12855155; doi:10.1093/ismeco/ycaf251)
Supplement: ycaf251_Supplemental_Files [file ycaf251_supplemental_files.zip › Table S1.docx]

**Table S1.** Ingredients and nutritional compositions of the forage-based (F) and grain-based (G) diets.

| Item | F | G |
| --- | --- | --- |
| **Ingredient composition, %, as-fed basis** |  |  |
| Alfalfa Hay | 17.9 | - |
| Corn Silage | 81.7 | 39 |
| Alfalfa Silage | - | 33.1 |
| Corn Grain | - | 27.5 |
| Limestone | 0.3 | - |
| Mineral | 0.1 | 0.1 |
| Salt | 0.1 | 0.1 |
| **Nutrient composition, DM basis** |  |  |
| Dry Matter, % | 50.7 | 52.7 |
| Acid Detergent Fiber, % | 21.78 | 15.63 |
| Total Digestible Nutrients, % | 73.51 | 79.87 |
| Metabolizable Energy, MJ kg^-1^ | 11.10 | 12.06 |
| Crude Protein, % | 13.1 | 12.3 |
| Calcium, % | 0.89 | 0.77 |
| Phosphorus, % | 0.45 | 0.42 |
| Magnesium, % | 0.42 | 0.34 |
| Potassium, % | 2.16 | 1.65 |
