# Supplementary figures and images for: Metatranscriptomics uncovers diet-driven structural, ecological, and functional adaptations in the rumen microbiome linked to feed efficiency
Source: ISME Commun. 2026 Jan 3;6(1):ycaf251. doi: 10.1093/ismeco/ycaf251 (PMC12855155; doi:10.1093/ismeco/ycaf251)

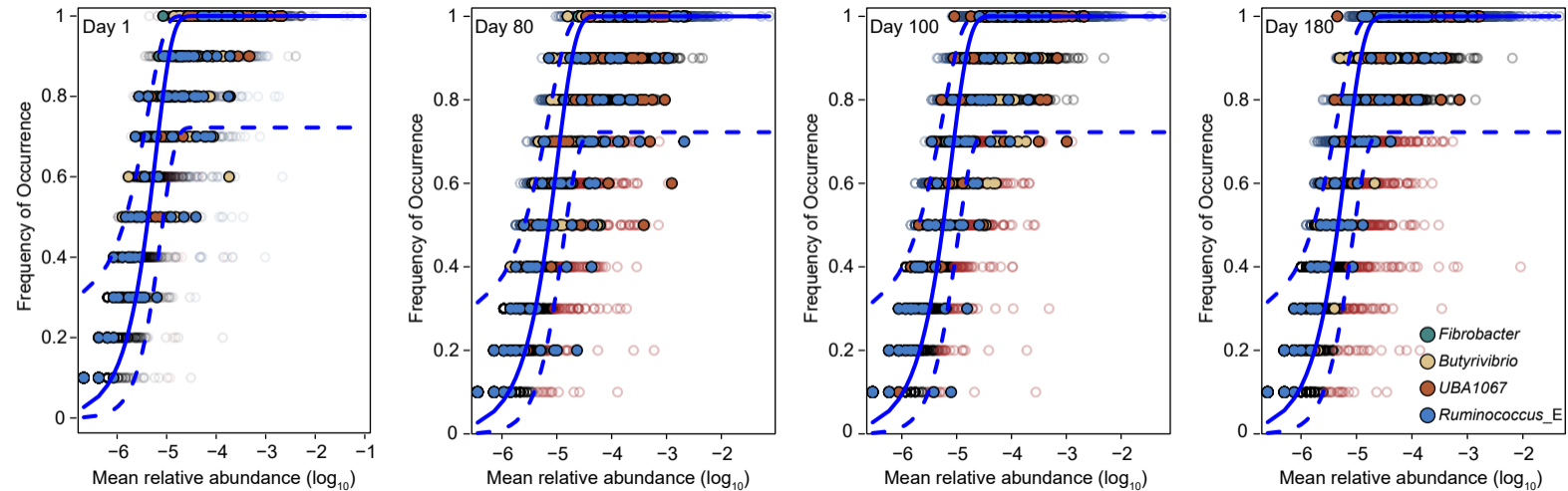

Supplement: ycaf251_Supplemental_Files [file ycaf251_supplemental_files.zip › Fig. S4.pdf]

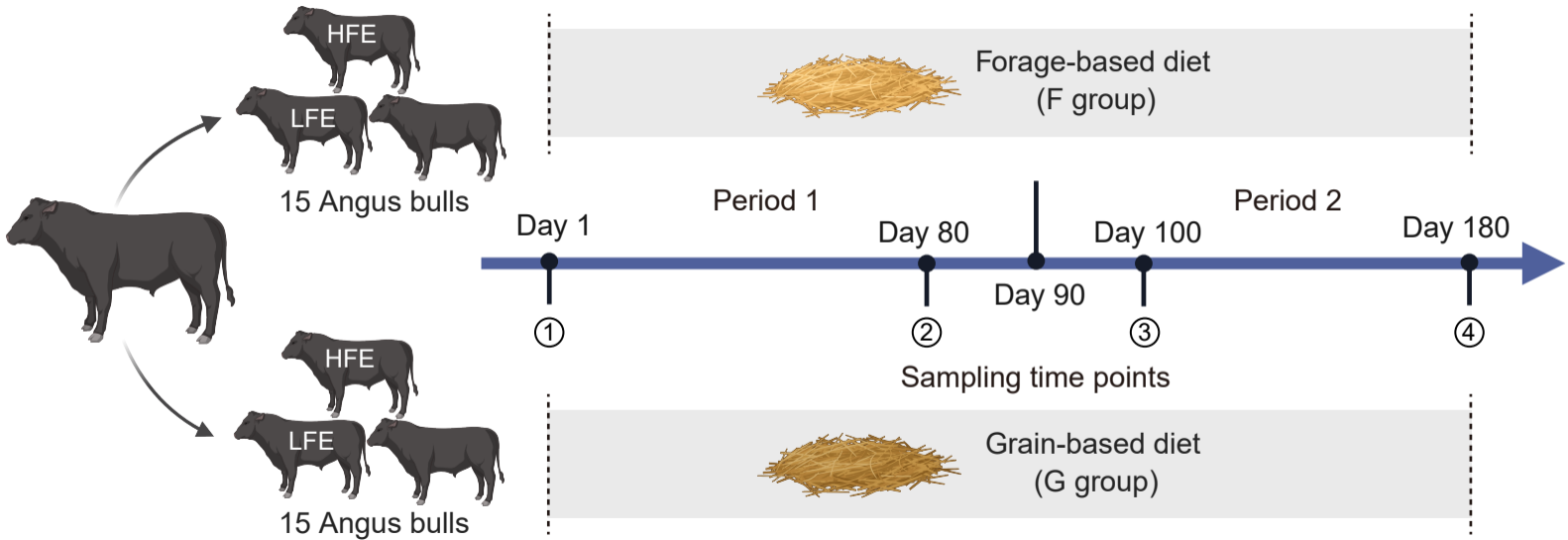

Supplement: ycaf251_Supplemental_Files [file ycaf251_supplemental_files.zip › Fig. S1.pdf]

CLR-transformed abundances

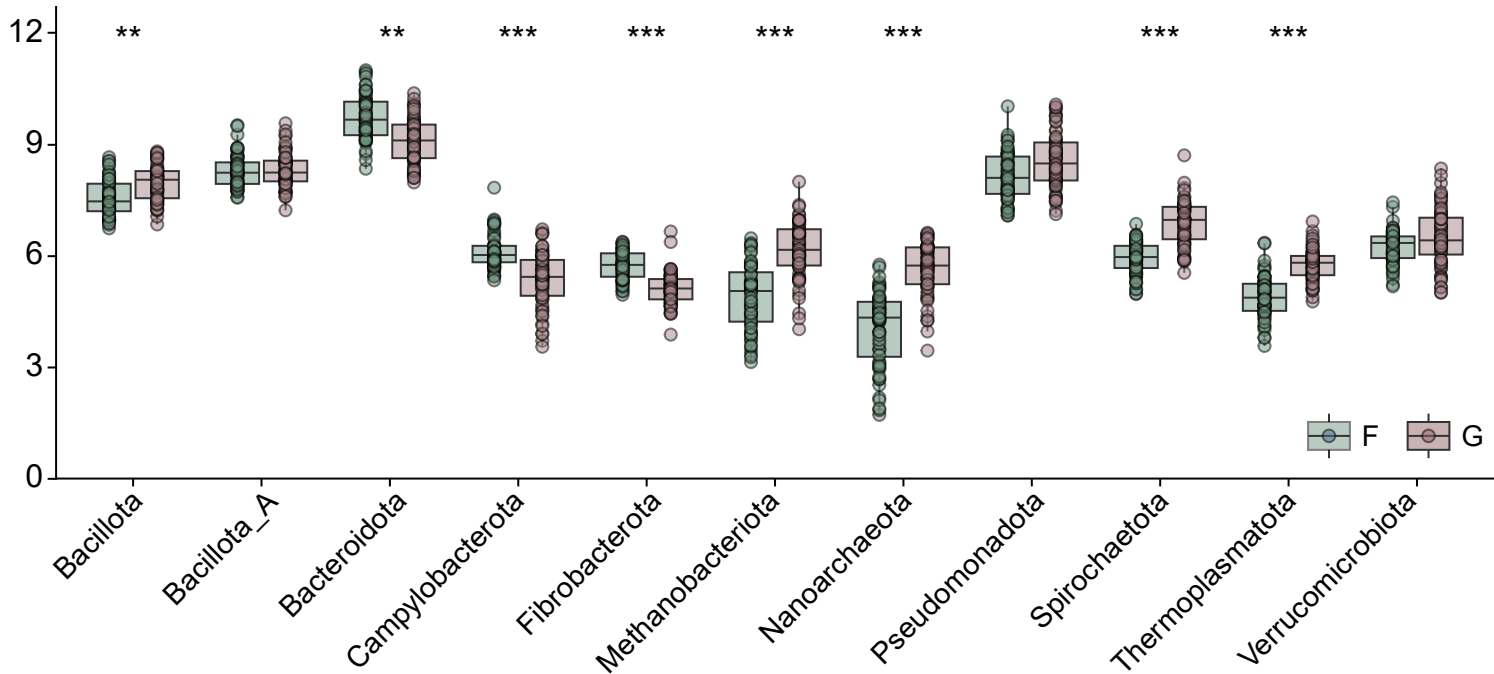

Supplement: ycaf251_Supplemental_Files [file ycaf251_supplemental_files.zip › Fig. S2.pdf]

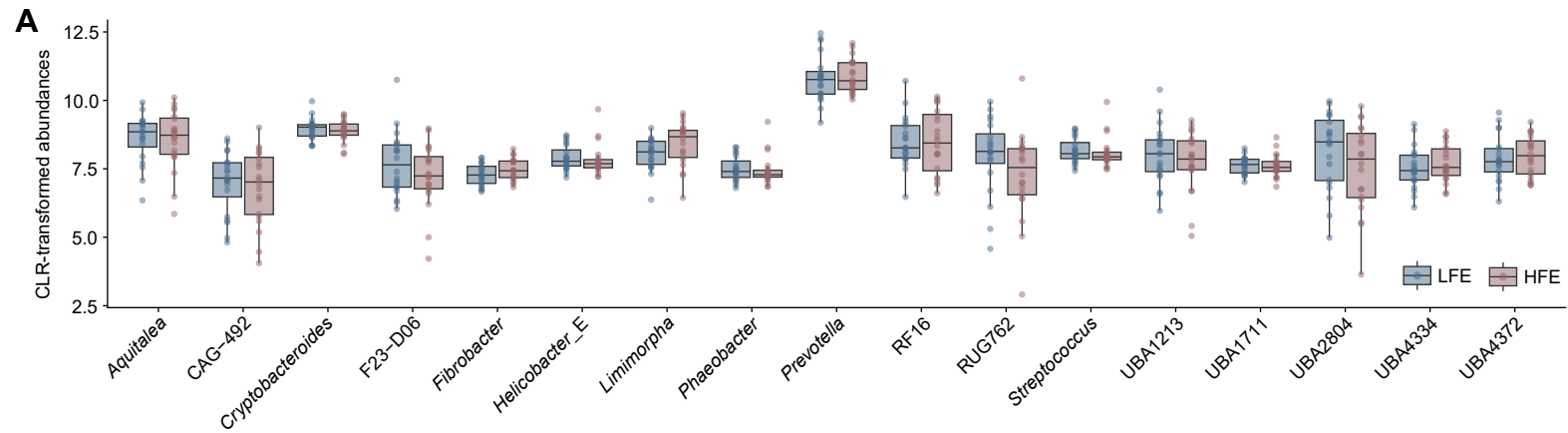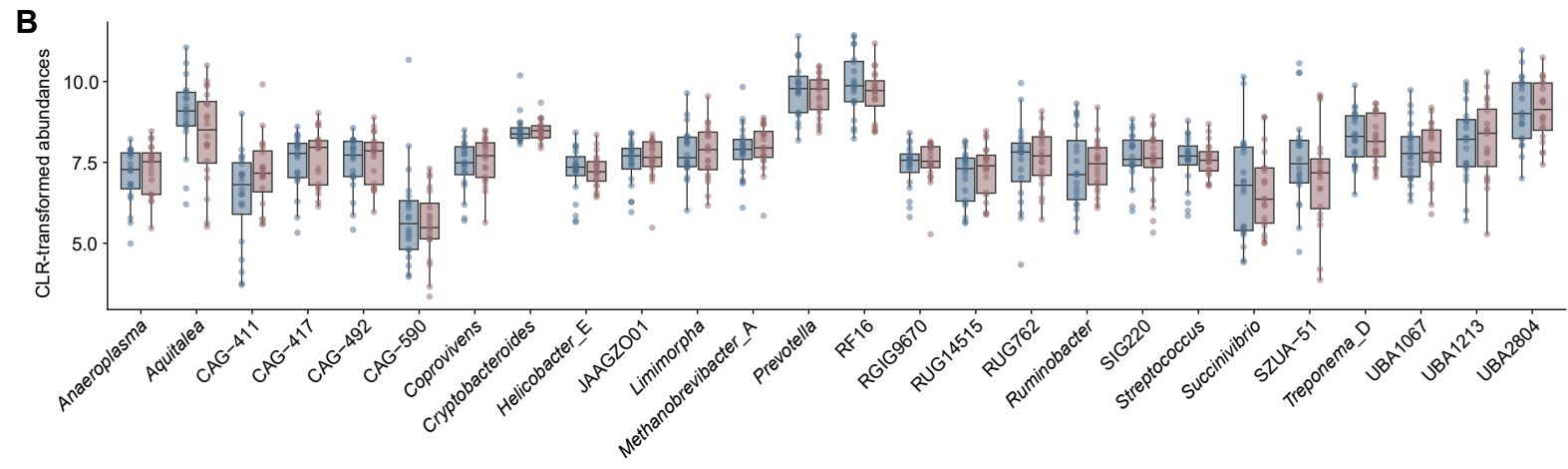

Supplement: ycaf251_Supplemental_Files [file ycaf251_supplemental_files.zip › Fig. S3.pdf]
